# Entropy Bounds for Hierarchical Molecular Networks

**DOI:** 10.1371/journal.pone.0003079

**Published:** 2008-08-28

**Authors:** Matthias Dehmer, Stephan Borgert, Frank Emmert-Streib

**Affiliations:** 1 Institute of Discrete Mathematics and Geometry, Vienna University of Technology, Vienna, Austria; 2 Department of Physics, University of Siegen, Siegen, Germany; 3 Department of Biomedical Sciences, Center for Cancer Research and Cell Biology, Queen's University Belfast, Belfast, United Kingdom; University of East Piedmont, Italy

## Abstract

In this paper we derive entropy bounds for hierarchical networks. More precisely, starting from a recently introduced measure to determine the topological entropy of non-hierarchical networks, we provide bounds for estimating the entropy of hierarchical graphs. Apart from bounds to estimate the entropy of a single hierarchical graph, we see that the derived bounds can also be used for characterizing graph classes. Our contribution is an important extension to previous results about the entropy of non-hierarchical networks because for practical applications hierarchical networks are playing an important role in chemistry and biology. In addition to the derivation of the entropy bounds, we provide a numerical analysis for two special graph classes, rooted trees and generalized trees, and demonstrate hereby not only the computational feasibility of our method but also learn about its characteristics and interpretability with respect to data analysis.

## Introduction

The investigation of topological aspects of chemical structures concerns a major part of the research in chemical graph theory and mathematical chemistry [1], [2], [3], [4]. Following, e.g., [5], [6], [7], [1], [2], [8], [9], classical and current research topics in chemical graph theory involve, e.g., modeling of chemical molecules by means of graphs, graph polynomials, graph-theoretical matrices, enumeration of chemical structures, and aspects of quantitative structure analysis like measuring the structural similarity of graphs and structural information. Further, a lot of the above mentioned contributions can be integrated under the following thematic categories which are well know in chemistry: QSAR and QSPR. QSAR (Quantitative structure-activity relationship) deals with descripting pharmacokinetic processes as well as biological activity or chemical reactivity [10], [11]. In contrast, QSPR (Quantitative Structure-Property Relationship) generally addresses the problem to convert chemical structures into molecular descriptors which are relevant to a physico-chemical property or a biological activity [11], [12]. However, a main problem in QSPR is to investigate relationships between molecular structure and physicochemical properties, e.g., the topological complexity of chemical structures [7], [13], [14], [11].

This paper mainly deals with a challenging problem of quantitative graph analysis: Deriving bounds for the entropies of hierarchical graphs. An important application area of information-theoretic methods applied to networks is, e.g., QSPR where our main focus lies on the examination of graph classes which are widely used in chemical graph theory and computational biology. Generally, there are two main directions in quantitative graph analysis: (i) Comparing and (ii) characterizing networks. Network comparison addresses the problem of measuring their structural similarity or distance, see, e.g., [15], [16], [17], [18], [19], [20], [21], [22]. In contrast, to characterize a network means that one has to infer structural network statistics which capture certain structural information of the networks [23], [24], [25], [26]. For giving a short review on information-theoretic methods to characterize graphs [6], [7], [14], [27], [28], [29], we want to emphasize that the problem of quantifying certain structural information of systems was a starting point of an emerging field that deals with applying information-theoretic techniques to networks, e.g., for investigating living systems [30], [31], [32], [33], [34], [35]. As a fundament, SHANNON [36] extended the concept of entropy that was known in thermodynamics for transmitting information. For this, he considered a message transmitted through information channels as a certain set of symbols denoted as an outcome which was selected from the ensemble of all *k* such sets containing the same total number of symbols *N* [27]. By assigning probabilities *p*_1_,*p*_2_,…,*p_k_* to each *i*-th outcome based on the quantities 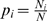 where *N_i_* denotes the number of symbols of the *i*-th outcome, SHANNON characterized the entropy *H* as the uncertainty of the expected outcome [27]. Then, the classical SHANNON-entropy formula to measure the average entropy of information per communication symbol can be expressed by
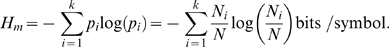
(1)
*H_m_* is often called the mean information. Additionally, BRILLOUIN [37] defined the total information as
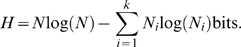
(2)Now, the topics we just mentioned [30], [31], [32], [33], [34], [35] have been mainly influenced by the, at that time, novel insight that an inferred or constructed graph structure can be considered as the result of a certain information process or communication between the elements of the underlying system [14], [36]. As a consequence [7], [38], Equation (1) and
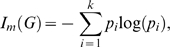
(3)Equation (2) can be now interpreted as the mean information content and the total information content
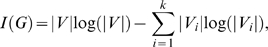
(4)of a graph *G*. Here, |*V*| denotes the number of vertices of a graph *G*, *k* denotes the number of different (obtained) sets of vertices, |*V_i_*| is the number of elements in the *i*-th set of vertices, and it holds 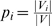.

The first attempt in this direction was given by [34] who developed a technique to determine the structural information content of a graph. This technique is based on the principle of finding distinguishable vertices of a graph to apply SHANNON's entropy (Equation (3) and Equation (4)) for determining the information content of such a graph-based system. Also, [38], [39], [40], [41] investigated this problem by using algebraic methods, i.e., determining the automorphism groups of graphs. We remark that the mentioned methods, e.g., [38], [39], [40], [41], [34], [35] for measuring the structural information content of a graph-based system are based on the following principle: Starting from a certain equivalence criterion, a graph-based system with *n* elements can be partitioned into *k* classes, see, e.g., [14]. As a consequence, a probability distribution can be obtained that leads directly to the definition of an entropy of the system under consideration (Equation (3) and Equation (4)). Following [14], [38], [28], the structural information content of such a system is interpreted as the entropy of the underlying graph topology. As a remark, we note that graph entropy definitions which are rooted in information theory can be found in [42], [43], [44], [45].

A major contribution of this paper addresses the problem of finding bounds for the entropies of hierarchical graphs, which often occurs in chemical graph theory and computational and systems biology. Here, the term “hierarchical” means that we deal with graphs having a distinct vertex that is called a root. To achieve this goal, we use an approach for determining the entropy of undirected and connected graphs that has been recently presented in [28]. In contrast to the classical methods which we have already outlined above, this method is based on assigning a probability value to each vertex in a graph by using a special information functional. The information functional we have presented in [28] is based on metrical properties of graphs, more precisely, on so-called *j*-spheres. In terms of practical applications, we want to point that the task of deriving bounds for the entropies of graphs is crucial because the exact entropy value can often not be calculated concretely, especially regarding large graphs. For this reason, entropy bounds for special graph classes help to reduce the complexity of such problems and can be also used for characterizing graphs or graph classes by using information-theoretic measures.

**Figure 1:**
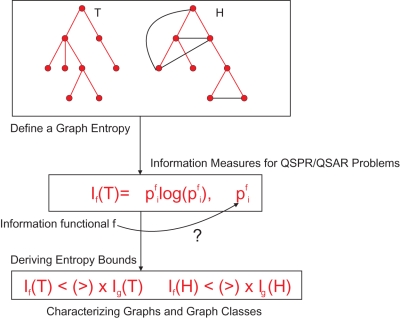
Overall approach to derive entropy bounds for hierarchical graphs.

As mentioned, hierarchical (rooted) graph structures do have a large application potential in chemical graph theory and computational biology. Therefore, we restrict our analysis on such graph structures. A further reason for focusing on rooted graphs is, to our knowledge, such a study does not exist. Another contribution of this paper deals with demonstrating the practical ability of the used graph entropy approach [28] by interpreting the produced numerical results. Starting from two graph classes, ordinary rooted trees and so-called generalized trees [46], [47], we show that our entropy measure captures important structural information meaningfully. To summarize the main contribution of this paper, Figure (1) shows the overall approach exemplarily.

## Analysis

### Applications of Hierarchical Graphs

In this section, we briefly outline some applications of hierarchical graphs in chemical graph theory and computational biology.

#### Mathematical Chemistry

There is a universe of problems dealing with trees for modeling and analyzing chemical structures [48], [1], [2], [3], [4]. However, also rooted tree structures are of particular interest because, e.g., considering such graph classes often helps to solve more general graph problems. In the following, we state some interesting applications of rooted trees in chemical graphs theory:

- Enumeration and coding problems of chemical structures by using rooted trees [49], [50], [51], [52].
- Describing so-called signatures as molecular descriptors for problems in QSAR [53].
- Graph polynomials of hierarchical graphs [54].
- Chemical graph analysis by using algebraic and metrical graph properties [55], [56], [57], [58].

#### Biology

Tree structures have been intensely investigated for solving and modeling biological problems. In particular, rooted trees often serve as an important graph representation for many biological classification problems as well as for problems in evolutionary biology [59]. To summarize some known approaches involving hierarchical graph structures, we state the following listing:

- Reconstruction problems and so-called supertree methods in phylogenetics [60], [61], [62], [63], [59].
- Modeling and analyzing RNA structures [64], [65].
- Supervised and unsupervised graph classification problems in computational biology [66], [67].
- Clustering problems in computational biology [68], [69].

### A Method for Determining the Entropy of Graphs

**Figure 2:**
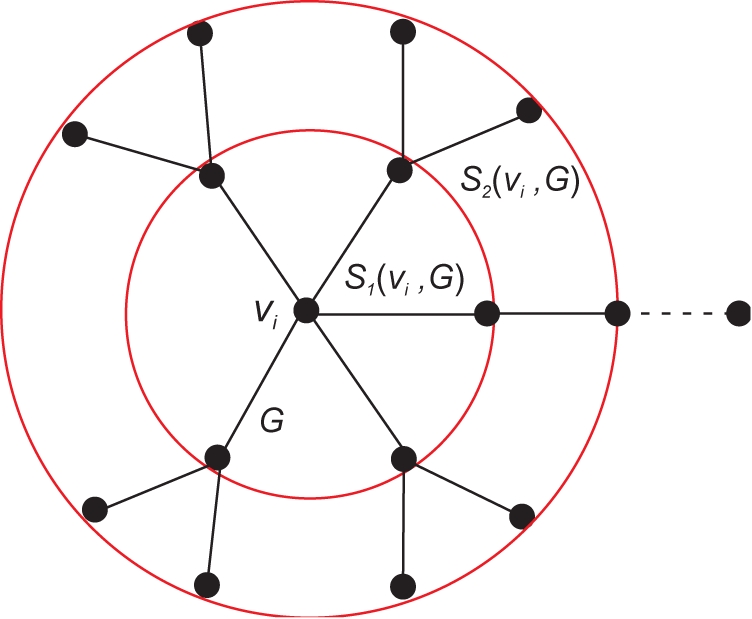
*G* represents an undirected and connected graph. For example, we get |*S*_1_(*v_i_*,*G*)| = 5 and |*S*_2_(*v_i_*,*G*)| = 9.

In this section, we briefly repeat the method to measure the entropy of arbitrary undirected and connected networks, see [28]. As mentioned, we will interpret and define the structural information content as the entropy of the underlying graph topology [28]. The method we want to use is mainly based on the principle to assign a probability value to each vertex in a graph by using a certain information functional for quantifying structural information in a graph and, hence, for determining its entropy. The information functional that has been used [28] is based on determining the so-called *j*-spheres of a graph. Before outlining the main construction steps of this approach, we want to mention that [70] also used so-called vertex distance degree sequences (DDS) to develop the idea of a graph center for chemical structures. Interestingly, the derived DDS-distributions correspond to vertex distributions by using *j*-spheres. Similarly to the just described idea, one main idea of the approach of [28] to determine the entropy of a graph was to use a connectivity concept to express neighborhood relations of its vertices. Finally, it turned out that a natural procedure for expressing such relations is to calculate the number of the first neighboring vertices, the number of the second neighboring vertices, etc. and, hence, this just corresponds to the definition of the *j*-sphere. As an example, Figure (2) shows the process of determining *j*-spheres visually.

In order to repeat the main construction step of the above mentioned graph entropy method, we first express some mathematical preliminaries [71], [72], [28]. We define an undirected, finite and connected graph by *G* = (*V,E*),|*V*|<∞, 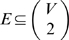. *G* is called connected if for arbitrary vertices *v_i_* and *v_j_* there exists an undirected path from *v_i_* to *v_j_*. Otherwise, we call *G* unconnected. *G_UC_* denotes the set of finite, undirected and connected graphs. The degree of a vertex *v*∈*V* is denoted by *δ*(*v*) and equals the number of edges *e*∈*E* which are incident with *v*. In order to measure distances between vertices in a graph, we denote *d*(*u,v*) as distance between *u*∈*V* and *v*∈*V* expressed as the minimum length of a path between *u,v*. We notice that *d*(*u,v*) is a metric. We call the quantity *σ*(*v*) = max*_u_*_∈*V*_ *d*(*u,v*) the eccentricity of *v*∈*V*. Further, *ρ*(*G*) = max*_v_*_∈*V*_ *σ*(*v*) is called the diameter of *G*. The *j*-sphere of a vertex *v_i_* regarding *G*∈*G_UC_* is defined as the set,
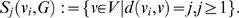
(5)Now, we state the definition of a special information functional that has been introduced in [28] to define the entropy of a graph. Here, the information functional *f^V^* quantifies structural information of a graph *G* by using the cardinalities of the corresponding *j*-spheres.

**Definition 2.1** *Let G*∈*G_UC_ with arbitrary vertex labels*. *For the vertex v_i_*∈*V*, *the information functional f^V^ is defined as*

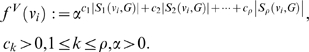
(6)
*f^V^* (*v_i_*) *captures structural information of G by using metrical properties of G. The parameters α and c_k_ are introduced to weight structural characteristics or differences of G in each sphere, e.g., a vertex with a large degree.*

As a remark, we generally see that it always
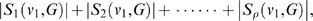
(7)

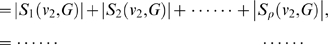
(8)

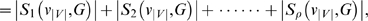
(9)holds [28]. Hence, the *c_k_* have to be chosen such that they are not equal, e.g, *c*_1_>*c*_2_>…>*c_ρ_*. Finally, we observe that the variation of *c_k_* and *α* aims to study the local information spread in a network.

**Definition 2.2** *The vertex probabilities are defined by the quantities*

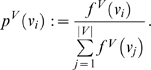
(10)


**Definition 2.3** *Let G* = (*V,E*)∈*G_UC_*. *Then, we define the entropy of G by*

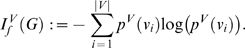
(11)


As outlined in [28], we recall that the process of defining information functionals and, hence, the entropy of a graph by using structural properties or graph-theoretical quantities is not unique. Consequently, each information functional captures structural information of a given graph differently. Further, we pointed out [28] that the parameter *α* can always be determined via an optimization procedure based on a given data set and, hence, is uniquely defined for a given classification problem.

### Bounds for the Entropies of Hierarchical Graphs

In this section, we derive bounds for the entropies of hierarchical graphs. For this, we use the entropy measure explained in the previous section. As mentioned, in this paper we choose the class of rooted trees and so-called generalized trees [47]. We notice that a generalized tree contains an ordinary rooted tree as a special case [47]. Further, it turned out that generalized trees can be very useful for solving current problems in applied discrete mathematics, computer science and systems biology [47], [73], [74], [66]. To start with the problem of finding entropy bounds, we first define the mentioned graph classes. Directed generalized trees have already been defined in [47].

**Definition 2.4** *An undirected graph is called undirected tree if this graph is connected and cycle free. An undirected rooted tree T* = (*V,E*) *is an undirected graph which has exactly one vertex r*∈*V for which every edge is directed away from the root r. Then, all vertices in T are uniquely accessible from r. The level of a vertex v in a rooted tree T is simply the length of the path from r to v. The path with the largest path length from the root to a leaf is denoted as h.*

**Definition 2.5** *As a special case of T* = (*V,E*) *we also define an ordinary w-tree denoted as T_w_ where w is a natural number. For the root vertex r, it holds δ*(*r*) = *w and for all internal vertices r*∈*V holds δ*(*v*) = *w*+1. *Leaves are vertices without successors. A w-tree is fully occupied, denoted by T_w_^o^, if all leaves possess the same height h.*

**Definition 2.6** *Let T* = (*V,E*_1_) *be an undirected finite rooted tree*. |*L*| *denotes the cardinality of the level set L*: = {*l*_0_,*l*_1_…,*l_h_*}. *The longest length of a path in T is denoted as h. It holds h* = |*L*|−1. Λ∶*V*→*L is a surjective mapping and it is called a multi level function if it assigns to each vertex an element of the level set L. A graph H* = (*V,E_GT_*) *is called a finite, undirected generalized tree if its edge set can be represented by the union E_GT_*: = *E*_1_∪*E*_2_∪*E*_3_, *where*

- *E*_1_ *forms the edge set of the underlying undirected rooted tree T.*
- *E*_2_ *denotes the set of horizontal Across-edges. A horizontal Across-edge does not change a level i.*
- *E*_3_ *denotes the set of edges which change at least one level.*

**Figure 3:**
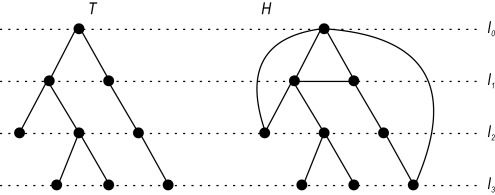
An undirected tree *T* and its corresponding undirected generalized tree *H*. It holds |*L*| = 4 and *h* = |*L*|−1 = 3.

As an example, Figure (3) shows an undirected rooted tree *T* and its corresponding undirected generalized tree *H*.

#### Entropy Bounds for Rooted Trees

Starting from the definition of the information functional *f^V^* (see Equation (6)), we first express a technical assertion proven in [75] that states a relationship between certain vertex probabilities. Starting from the definition of *f^V^*, this assertion expresses that it is always possible to infer inequalities between the corresponding vertex probabilities. In order to achieve this, we also use simple estimations of parameters which we introduce in Lemma (2.1). Finally, we will see that by applying this lemma, we can easily derive entropy bounds for the graph classes under consideration. Hence, the following lemma serves as a fundament for the proofs of some theorems we want state in this section.

**Lemma 2.1** *Let T be a rooted tree with a certain height h and let f^V^ be the information functional represented by* *Equation (6)* *. Further, we define the quantities*

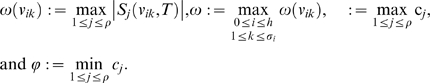
(12)
*It holds*

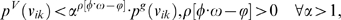
(13)
*where*

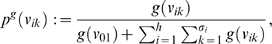
(14)
*and* 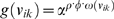. *p^V^* (*v_ik_*) *denotes the vertex probability of v_ik_ regarding f^V^. Further, v_ik_ denotes the k-th vertex on the i-th level*, 1≤*i*≤*h*,1≤*k*≤*σ_i_*. *σ_i_ denotes the number of vertices on level i.*

In the following, we derive entropy bounds for hierarchical networks by applying Lemma (2.1). Because Lemma (2.1) provides inequalities between vertex probabilities for each vertex in a graph, the main idea for inferring entropy bounds is to add up the obtained inequalities. As a result, we get relations between graph entropy measures for hierarchical networks which can be interpreted as entropy bounds. Also, the conclusion of Lemma (2.1) implies that by varying the Inequalities (13), special entropy bounds can be obtained.

**Theorem 2.2** *Let T be a rooted tree. For the entropy of T, it holds the inequality*

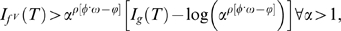
(15)
*where*

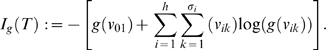
(16)


**Proof**: To start the proof, we consider Inequality (13) in Lemma (2.1). If we multiply this inequality by -1, we get
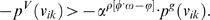
(17)Now, by using the assertion of Lemma (2.1) and the monotonicity property of the logarithm function, we obtain
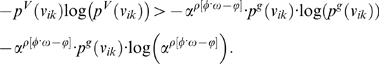
(18)If we perform this step for each vertex *v_ik_*∈*V* and then add up the obtained inequalities, we get
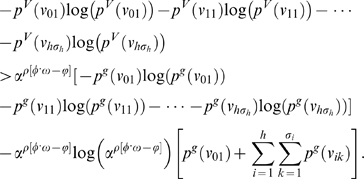
Because by definition it holds
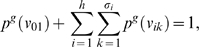
we obviously get
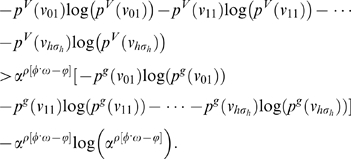
(19)Now, by using the definition of the graph entropy (see Definition (2.3)), Inequality (19) finally becomes to
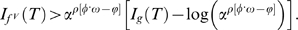
This completes the proof of the theorem.

By considering special classes of rooted trees, we obviously get special bounds for the corresponding entropies.

**Theorem 2.3** *Let T_w_^o^ be a fully occupied w-tree. For the graph entropy of T_w_^o^ holds*

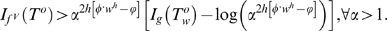
(20)


**Proof**: Let *T_w_^o^* be a fully occupied *w*-tree. Therefore, it holds *ρ* = 2*h*. Starting from the root vertex *v*_01_, all other vertices are reachable. Hence, we obtain |*S_h_*(*v*_01_,*T_w_^o^*)| = *w^h^*. Then, we clearly get |*S_j_*(*v_ik_,T_w_^o^*)|<*w^h^*, 1≤*j*≤2*h*. Hence, we can set *ω* = *w^h^*. Now, the proof of the Theorem (2.3) can be obtained by analogously applying the same technique and steps of the proof of Theorem (2.2).

**Theorem 2.4** *Let T_w_ be an ordinary w-tree. For the graph entropy of T_w_ holds*

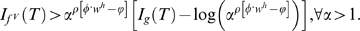
(21)


**Proof**: Let *T_w_* be an ordinary *w*-tree. Actually, it holds *ω*≤*w^h^*. From this, and by applying Lemma (2.1), we yield
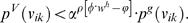
(22)Finally, we obtain the assertion of the theorem by applying the same technique and steps performed in the proof of Theorem (2.2).

We emphasize that each information functional captures structural information of a graph differently. Obviously, the resulting graph entropies are also different. If we now apply Theorem (2.2) and additionally assume an abstract information functional *f* ^*^, we find as a consequence of the previous theorems that one can infer a statement that expresses a relationship between the resulting graph entropies. These kind of inequalities can be used to study the influence of an information functional on the final graph entropies.

**Corollary 2.5** *Let T be a rooted tree and let f* ^*^(*v_ik_*) *be an information functional such that*

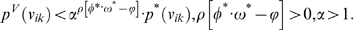
(23)
*p^V^* (*v_ik_*) *and p* ^*^(*v_ik_*) *denotes the vertex probability value* (*k-th vertex on the i-th level*) *regarding f^V^ and f* ^*^. *Then, it holds*

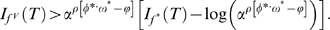
(24)


#### Entropy Bounds for Generalized Trees

In this section, we give a first attempt to state entropy bounds for certain classes of generalized trees. By only allowing generalized trees with specific edge sets, we get bounds for the entropies of special classes of generalized trees. The assertion of the next theorem means the following: The entropy of a specific generalized tree can be characterized by the entropy of another generalized tree that is extremal with respect to a certain structural property.

**Theorem 2.6** *Let H* = (*V,E_GT_*) *be a generalized tree with E_GT_*: = *E*_1_∪*E*_2_, *i.e., H possesses Across-edges only. Starting from H, we define H* ^*^ *as the generalized tree with the maximal number of Across-Edges on each level i*,1≤*i*≤*h*.

- *First, there exist positive real coefficients c_k_ which satisfy the inequality system*

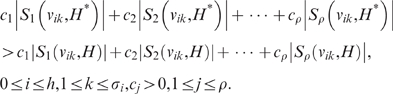
(25)
- *Second, it holds*

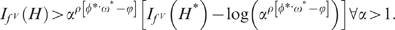
(26)

**Proof**: We assume *H* = (*V,E_GT_*) such that *E_GT_* = *E*_1_∪*E*_2_. Besides edges *e*∈*E*_1_, *H* possesses Across-edges *e*∈*E*_2_ only. Then, we first determine
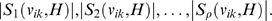



Now, we consider *H* ^*^ and find that the total number of Across-edges for each level equals 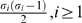. Except for the root vertex *v*_01_, we further see that in particular |*S*_1_(*v_ik_*,*H* ^*^)|≥|*S*_1_(*v_ik_*,*H*)| holds. This corresponds to the fact that *H* ^*^ has normally more connections than *H*. Finally, the cardinalities of the remaining *j*-spheres of *H* ^*^ increase correspondingly. Therefore, we conclude that we can find coefficients *c_k_*>0 such that the Inequality System (**25**) holds. But from this, we directly obtain
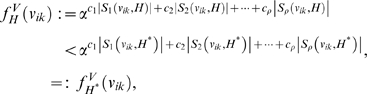
(27)if *α*>1, 1≤*i*≤*h*, 1≤*k*≤*σ_i_*. *f_H_^V^* (*v_ik_*) and 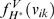 denotes the information functional *f^V^* regarding *H* and *H* ^*^, respectively. We want to emphasize that it holds 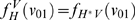. Similarly as in Lemma (2.1), by using the quantities we yield
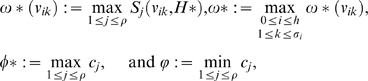


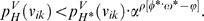
(28)Finally, Equation (26) can be obtained by applying the assertion of Theorem (2.2).

We want to remark that by using the main argument of Theorem (2.6), one can easily express similar assertions for other specific generalized tree classes. To finalize this section, we state a simple lemma concerning the maximum entropy of a graph. Then, we apply this assertion to generalized trees.

**Lemma 2.7** *Let K*_|*V*|,|*V*|_ *be the complete graph with* |*V*| *vertices*. *K*_|*V*|,|*V*|_ *maximizes the graph entropy with respect to the information functional f^V^*, *i.e.*,
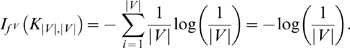
(29)


**Theorem 2.8** *Let H* = (*V^H^,E*) *be an arbitrary generalized tree and let H*_|*V*|,|*V*|_ *be the complete generalized tree such that* |*V^H^*|≤|*V*|. *It holds*

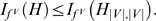
(30)


**Proof**: The proof follows directly by using the monotonicity property of the logarithm function and the assertion of Lemma (2.7).

**Corollary 2.9** *Let H* ^*^ = (*V* ^*^,*E* ^*^) *and it holds* |*V* ^*^|≤|*V*|.
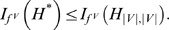
(31)


## Results and Discussion

### Numerical Results for Hierarchical Graphs

This section aims to demonstrate that our entropic measure is able to distinguish certain graph classes of hierarchical graphs structurally by comparing the resulting cumulative entropy distributions. As a result of our numerical analysis, we will find that the calculated entropy distributions can be clearly distinguished and, hence, also the graph classes under consideration. Thus, this proves that the entropy measure captures significant structural information. To start, we give a short overview on the key steps we performed to carry out our numerical analysis:

- Generate the data classes *C_α_^RT^* and *C_α_^GT^*. For this, we randomly create rooted trees with a fixed height *h*. Further, we use these trees to generate generalized trees (see also below).
- Choose the parameters *c_k_*.
- Vary *α* to compute *I_f_^V^* for different classes *C_α_^RT^* and *C_α_^GT^*.
- Compute the mean of the entropies for each such class denoted by μ and the variances *σ* ^2^.
- Compute and interpret the cumulative entropy distributions for *C_α_^RT^* and *C_α_^GT^*.

We remark that the intuitive meaning of the entropy *I_f_^V^* (*G*) has been already explained in [28]. Now, we start our numerical section with defining some data classes. These data classes emerge from starting with fixed sets of hierarchical graphs and by varying certain parameters.

**Definition 3.1** *The class C_α_^RT^ denotes a certain set of rooted trees whose entropies have been computed by using the value α and the coefficient vector* (*c*_1_,*c*_2_,…,*c_ρm_*). *We set*

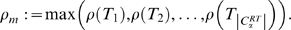

*Correspondingly, C_α_^GT^ denotes a certain set of generalized trees whose entropies have been computed by also using the value α and* (*c*_1_,*c*_2_,…,*c_ρm_*).

**Figure 4:**
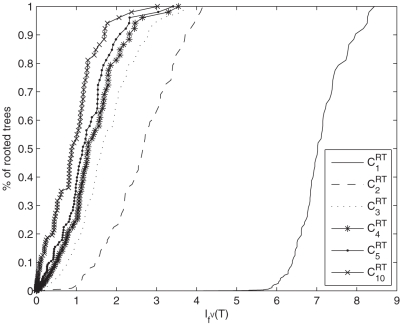
Cumulative entropy distributions of the classes *C_α_^RT^* for *h* = 8. The *x*-axis corresponds to the entropy *I_f_^V^* (*T*) and the *y*-axis represents the cumulative entropy distribution for *C*_1_ *^RT^* -*C*_5_ *^RT^* and *C*_10_ *^RT^*.

**Figure 5:**
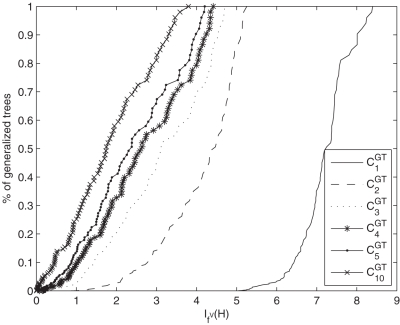
Cumulative entropy distributions of the classes *C_α_^GT^* for *h* = 8. The *x*-axis corresponds to the entropy *I_f_^V^* (*H*) and the *y*-axis represents the cumulative entropy distribution for *C*_1_ *^GT^* -*C*_5_ *^GT^* and *C*_10_ *^GT^*.

In order to compute the graph entropies concretely, we choose the *c_k_* values such that
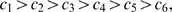
holds, and set *c*_1_: = 6,*c*_2_: = 5,*c*_3_: = 4,*c*_4_: = 3,*c*_5_: = 2,*c*_6_: = 1. A class *C_α_^RT^* was generated by providing a fixed value *h* as the height of each tree 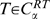. Further, each 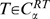 has an unique root vertex and the remaining vertices and edges were created randomly. To generate a class *C_α_^GT^*, we first compute an arbitrary random tree with height *h* as mentioned and, then, a certain number of additional edges of a generalized tree randomly. The numerical results of our study are summarized in Table (1). As we have already mentioned, we computed the entropies of certain classes of rooted and generalized trees with a fixed height *h* by varying the *α*-value. We notice that by providing a fixed height *h*, the number of vertices of *T* or *H* can be nevertheless extremely different. Now, from Table (1) we see that the resulting entropies of generalized trees are in average larger than the entropies of rooted trees, depending on *α*. This corresponds to our intuition that a generalized tree can be generally considered as structurally more complex than an ordinary rooted tree. To argue in this way, we apply a definition due to [11] that states, the higher the information content (entropy) of a system is, the more complex is the system. Further, one finds that the variances of the generated tree and generalized tree classes can be clearly distinguished. This can be also explained by the fact that a set of generalized trees is in average more structurally complex and diverse than a set of rooted trees with the same height *h*. Also, we observe that the larger the *α*-value of *C_α_^RT^* and *C_α_^GT^* is, the smaller is the resulting mean and variance. Additionally, we also find that the entropy of a graph decreases with an increasing *α*-value. In the following, we interpret the cumulative entropy distributions (for *h* = 8) which are shown in Figure (4) and Figure (5). Such a distribution expresses the percentage rate of graphs (of the cardinality of *C_α_^RT^* or *C_α_^GT^*) which possess an entropy value less or equal *I_f_^V^* (*T*) or *I_f_^V^* (*H*). As an important observation, we find that for *α*∈{2,3,4,5,10} the cumulative entropy distributions of *C_α_^RT^* (see Figure (4)) are clearly different from the corresponding cumulative distributions of *C_α_^GT^* (see Figure (5)). Hence, we interpret this result such that the entropy measure (by incorporating the information functional *f^V^*) is able to detect that we deal with different graph classes. The reason why the distribution for *C*_1_ *^RT^* and *C*_1_ *^GT^* seems to be almost equal is related to the fact that our entropy measure has always a maximum at *α* = 1. For this case, the entropies of trees- and generalized trees are almost equal. We remark that we have already been proven that the entropy functional (by using *f^V^*) possesses for every graph a maximum at *α* = 1, see [28]. As the main result of this section, we find that our entropy measure captures important structural information meaningfully and, hence, detects that rooted and generalized trees manifest structurally different graph classes.

**Table 1:** μ represent the means of the entropies for each class C_α_^RT^ and C_α_^GT^ and σ^2^ denotes the corresponding variance.

h = 7										
	C_1_ *^RT^*	C_1_ *^GT^*	C_2_ *^RT^*	C_2_ *^GT^*	C_3_ *^RT^*	C_3_ *^GT^*	C_4_ *^RT^*	C_4_ *^GT^*	C_5_ *^RT^*	C_5_ *^GT^*
μ	6.029	6.218	1.766	2.846	1.123	1.987	0.895	1.629	0.775	1.423
σ^2^	0.595	0.614	0.828	1.221	0.611	1.110	0.506	0.955	0.444	0.841
	C_6_^RT^	C_6_^GT^	C_7_^RT^	C_7_^GT^	C_8_^RT^	C_8_^GT^	C_9_^RT^	C_9_^GT^	C_10_^RT^	C_10_^GT^
μ	0.701	1.287	0.649	1.188	0.610	1.113	0.580	1.054	0.556	1.005
σ^2^	0.402	0.758	0.373	0.696	0.351	0.648	0.333	0.611	0.320	0.580

h = 8										
	C_1_^RT^	C_1_^GT^	C_2_^RT^	C_2_^GT^	C_3_^RT^	C_3_^GT^	C_4_^RT^	C_4_^GT^	C_5_^RT^	C_5_^GT^
μ	6.713	6.767	2.087	3.434	1.287	2.389	1.009	1.959	0.867	1.720
σ^2^	0.546	0.672	1.027	1.571	0.711	1.792	0.581	1.686	0.511	1.559
	C_6_^RT^	C_6_^GT^	C_7_^RT^	C_7_^GT^	C_8_^RT^	C_8_^GT^	C_9_^RT^	C_9_^GT^	C_10_^RT^	C_10_^GT^
μ	0.780	1.566	0.721	1.456	0.677	1.372	0.643	1.306	0.616	1.253
σ^2^	0.467	1.449	0.436	1.355	0.412	1.277	0.394	1.211	0.379	1.153

It holds |C_α_^RT^| = |C_α_^GT^| = 100. α varies in natural numbers from 1 to 10, the step size is equal to 1.

### Summary and Conclusion

In this paper, we investigated the problem of finding entropy bounds for hierarchical graphs. Based on an entropic measure to determine the entropy of graphs, we derived certain estimations for the corresponding entropies. We now summarize the main contributions and arguments of our paper as follows:

We defined two classes of hierarchical graphs, rooted trees and generalized trees. A generalized tree is structurally more complex than an ordinary rooted tree because it contains a rooted tree as a special case. As a main result, we proved entropy bounds for rooted trees as well as for generalized trees. Also, assuming specific structural properties of the graph classes under consideration led us to characteristic bounds. It is important to note that we presented only one method for finding those entropy bounds, different bounds can be derived by using different entropy measures and techniques. To classify these bounds, we call the derived bounds *implicit bounds* because the entropy of a graph was estimated by a quantity that contains another graph entropy expression. Generally, bounds to estimate the entropy of graphs are very useful for practical applications because the real entropy value is often difficult to obtain. Particularly, an interesting result represents Corollary (2.5). From this assertion, we found that an information functional (e.g., *f^V^* or *f* ^*^) has an influence on the resulting graph entropy because each such functional quantifies structural information differently. Hence, Corollary (2.5) can be used for describing relations of the resulting entropies by using different information functional.

Further, we performed a numerical study to demonstrate the practical ability of our graph entropy measure. Based on two generated graph classes of rooted and generalized trees, we computed the entropies of each such class by varying the free parameter *α*. Then, we calculated the cumulative entropy distributions for these classes. From the obtained results we could conclude that our entropy measure can distinguish between rooted trees and generalized trees. This implied that the used entropy measure captures significant structural information because we know that rooted trees and generalized trees are different graph classes.
